# Abs No: HLA-A*31:01 positive hypersensitive patient

**DOI:** 10.1186/2045-7022-4-S3-P117

**Published:** 2014-07-18

**Authors:** Maike Lichtenfels, John Farrell, Monday Ogese, Catherine Bell, Sidonia Eckle, James McCluskey, Kevin Park, Ana Alfirevic, Dean Naisbitt, Munir Pirmohamed

**Affiliations:** 1 University of Liverpool, MRC Centre for Drug Safety Science, UK; 2 University of Melbourne, Department of Microbiology & Immunology, Australia

## Background

Hypersensitivity reactions to carbamazepine (CBZ) have been shown to be strongly associated with specific human leukocyte antigen (HLA) alleles, with carriers of the HLA alleles presenting an increased risk of developing hypersensitivity. HLA-B*15:02 was detected in almost all cases of CBZ-induced Stevens-Johnson syndrome (SJS) in patients of Han Chinese or South-East Asian ancestry, and its functional role in CBZ-induced SJS has been well characterised. HLA-A*3101 is associated with all clinical phenotypes of CBZ-induced hypersensitivity in Caucasian and Japanese patients. However, functional studies investigating the role of HLA-A*31:01 in CBZ-specific T-cell responses have not been performed. Furthermore, CBZ-specific T-cells of CD4+ and CD8+ phenotype are readily detectable in Caucasian patients, which is in stark contrast to the dominant CD8+ T-cell response in Han Chinese. In this study we therefore investigated the HLA restriction of CBZ-reactive T-cells from a HLA-A*31:01 positive CBZ hypersensitive patient, focusing on both the CD4+ and CD8+ cells.

## Method

Drug-specific T-cell clones (TCC) were generated by serial dilution, and their reactivity to CBZ measured by proliferation assay. HLA restriction of TCC was assessed using anti-HLA antibodies as well as allogeneic HLA-mismatched antigen-presenting cells (APC).

## Results

Activation of CD8+ TCC could be blocked by an anti-HLA class I antibody, and also when an anti-HLA A30/A31 antibody was used. Using allogeneic HLA-A*31:01+ APC and control APC expressing common HLA-A alleles, a CBZ-specific HLA-A*31:01 dependent activation of CD8+ TCC could be demonstrated. CBZ reactivity of CD4+ TCC was restricted by HLA class II, predominantly HLA-DR and HLA-DP. CD4+ TCC proliferated in the presence of CBZ and APC expressing HLA-DRB1*04:04, an HLA class II allele known to be part of a common haplotype with HLA-A*31:01 in Caucasians. APCs expressing other HLA-DRB1 alleles were unable to stimulate a response.

## Conclusion

We were able to characterise the individual HLA restriction profile of CBZ-specific CD4+ and CD8+TCC from an HLA-A*31:01+ patient. We discovered an HLA class II allele, i.e. HLA-DRB1*04:04, to be functionally important for the activation of CD4+ T-cells. The strong linkage disequilibrium between the two alleles suggests a common haplotype may contribute to the multi-clonal response seen in Caucasian patients hypersensitive to CBZ.

